# Screening Epitopes Through Comparative Analysis of Children and Mice Immune Responses to Pertussis Toxin Subunits (S1–S5) Induced by Whole-Cell Pertussis Vaccination

**DOI:** 10.3390/vaccines14050413

**Published:** 2026-05-02

**Authors:** Salvatore Giovanni De-Simone, Guilherme Curty Lechuga, Paloma Napoleão-Pêgo, Mariana Silva Freitas, Sergian Vianna Cardozo, Carlos Medicis Morel, David William Provance Jr, Flavio Rocha da Silva

**Affiliations:** 1 Center for Technological Development in Health (CDTS), Oswaldo Cruz Foundation, Rio de Janeiro 21040-900, Brazil; guilherme.curty@fiocruz.br (G.C.L.); paloma.pego@fiocruz.br (P.N.-P.); msfreitas@id.uff.br (M.S.F.); carlos.morel@fiocruz.br (C.M.M.); bill.provance@fiocruz.br (D.W.P.J.); flavio.rocha@ioc.fiocruz.br (F.R.d.S.); 2 Program of Post-Graduation on Science and Biotechnology, Department of Molecular and Cellular Biology, Biology Institute, Federal Fluminense University, Niteroi 22040-036, Brazil; 3 Program of Post-Graduation on Parasitic Biology, Oswaldo Cruz Institute, Oswaldo Cruz Foundation, Rio de Janeiro 21040-900, Brazil; 4 Program in Translational Biomedicine (BIOTRANS), Graduate Department of Health, University of Grande Rio (UNIGRANRIO), Caxias 25071-202, Brazil; sergian.cardozo@unigranrio.edu.br

**Keywords:** *Bordetella pertussis*, Pertussis toxin S1–S5 subunits, infants and mice epitope mapping, peptide array, SPOT-synthesis

## Abstract

Background: Pertussis toxin (Ptx) is a major virulence factor and protective antigen of *Bordetella pertussis*. Understanding its antigenic landscape is essential for improving vaccine design. This study aimed to compare the linear epitope profiles of Ptx recognized by antibodies from vaccinated children and mice, identifying conserved and species-specific immune targets across subunits S1–S5. Methods: Two libraries of overlapping 14-mer peptides spanning the full-length Ptx sequence were synthesized. Sera from children and mice immunized with the whole-cell pertussis vaccine were analyzed to map antibody-binding regions. Comparative and structural analyses were performed to evaluate epitope distribution and recognition patterns. Results: Murine sera recognized 12 major epitopes, whereas children’s sera identified 24. Eleven epitopes were shared between species, mainly in subunits S1 (Ep3–5, 7, 9, 10), S3 (Ep20, 21, 25, 26), and S5 (Ep32), although minor positional shifts were observed. Eight epitopes were unique to children’s sera, located in S1 (Ep1, 6, 8), S3 (Ep22–24), and S4 (Ep27, 29–30). In the S2 subunit, four distinct epitopes were identified for each species, while only one mouse-specific epitope was detected in S4 (Ep28). Structural analysis revealed non-uniform antibody recognition, with dominant targeting of S3 and conserved antigenic hotspots, as well as selective recognition of the catalytic S1 subunit. Fourteen novel epitopes were identified. Conclusions: These findings highlight both shared and species-specific Ptx epitopes, revealing differences between murine and human immune responses. The identified conserved regions and novel epitopes provide a basis for improved pertussis vaccine design.

## 1. Introduction

Pertussis is an acute respiratory disease caused by the Gram-negative bacterium *Bordetella pertussis*, with high morbidity and mortality among newborns and young children. Pertussis toxin (Ptx) plays a central role in disease pathogenesis and is a major immunogen, serving as a key antigen in both whole-cell (wP) and acellular (aP) pertussis vaccines [1,2]. Comparative analyses of immune responses to Ptx in mice and humans provide important insights into host-specific mechanisms of protection, antibody repertoire diversity, and the translational relevance of animal models [3]. Although both species develop antibody-mediated immunity following infection or vaccination, significant differences exist in the specificity, magnitude, and persistence of these responses [4,5,6].

In mice, immunization with whole-cell DTP vaccines induces a robust polyclonal response targeting multiple Ptx subunits (S1–S5) and other bacterial antigens, such as filamentous hemagglutinin and pertactin [7]. This response is predominantly characterized by IgG2a and IgG2b subclasses, reflecting a Th1/Th17-skewed profile associated with efficient opsonization and complement activation [5,8,9]. Mice can also generate mucosal IgA, particularly following whole-cell or intranasal immunization, contributing to toxin neutralization at the site of infection [10,11,12,13]. However, murine antibody responses tend to preferentially target linear and conserved epitopes, especially within surface-exposed regions of the B oligomer (S2–S4), with comparatively limited recognition of the enzymatic S1 subunit [14,15,16,17,18].

In contrast, children develop strong humoral responses to Ptx following infection or vaccination, but with distinct patterns of immunodominance [19,20]. Acellular vaccines typically induce IgG1 and IgG4 responses consistent with a Th2-biased profile, whereas whole-cell vaccines elicit a more balanced IgG1/IgG3 response associated with enhanced neutralizing capacity [6]. Importantly, human antibodies frequently recognize conformational epitopes spanning multiple subunits, including the enzymatic domain of S1 and receptor-binding regions of S2 and S3 [21,22]. This suggests a greater reliance on tertiary and quaternary structural determinants compared to the predominantly linear epitope recognition observed in murine systems.

Functionally, antibodies from both species can neutralize Ptx; however, differences exist in the durability and magnitude of the response. In children, antibody titers and immunological memory tend to be more sustained, particularly following booster vaccination or natural infection [23,24]. In contrast, murine responses peak rapidly but decline more quickly, reflecting a shorter-lived plasma cell response [25,26]. Despite these differences, murine models remain essential for mechanistic studies and for the identification of linear B-cell epitopes relevant to vaccine-induced immunity.

In this context, the identification of novel epitopes is critical for expanding the repertoire of immunogenic targets, particularly those located in conserved and functionally relevant regions. Such epitopes may enhance vaccine efficacy by promoting broader and more durable immune responses, including both antibody and T-cell-mediated immunity, while mitigating the impact of antigenic variation. Moreover, understanding interspecies differences in epitope recognition is essential for translating findings from animal models to human immunity and for guiding the rational design of next-generation pertussis vaccines.

Here, we performed a comprehensive epitope mapping analysis of Ptx, focusing on the PtxS1 subunit. Our results identify ten previously underexplored linear epitopes that may contribute to immune recognition of the toxin. Although linear mapping does not fully capture the conformational determinants critical for neutralization, these epitopes may represent accessible or processing-derived regions that can elicit immune responses. Notably, conserved epitopes within functionally important domains of PtxS1 may serve as promising targets for developing improved vaccine strategies that incorporate both dominant and subdominant antigenic determinants.

## 2. Materials and Methods

### 2.1. Immunization of Mice

Thirty-eight NIH Swiss mice (12–16 g; 21–28 days old) were immunized intraperitoneally with a whole-cell pentavalent vaccine (DTP-HepB/Hib; Instituto Butantan and Bio-Manguinhos/Fiocruz, Brazil). The vaccine was reconstituted in sterile saline and administered in 0.5 mL doses containing 2 IU (as defined by the Brazilian National Immunization Program) at 21-day intervals. Blood samples were collected one week after the final immunization, the sera were separated, stored at −20 °C, and pooled prior to use [27]. All animal procedures were performed in accordance with the Guidelines for the Use of Animals in Biochemical Research and were approved by the institutional ethics committee (CEUA protocol no. 052/2021).

### 2.2. Human Sera

Ninety-two children aged 1–12 years (median age 7.5 years), vaccinated intramuscularly with the pentavalent whole-cell DTP (DTP-HepB/Hib; Butantan e Bio-Manguinhos, Rio de Janeiro, Brazil) vaccine and with no evidence of acute infection or a known history of whooping cough or diphtheria, were enrolled in this study. The vaccination and blood test collection (15 days later, the single immunization) were performed at Ismélia da Silveira Municipal Children’s Hospital (Duque de Caxias, Rio de Janeiro, Brazil) as part of the Ministry of Health’s 2024 annual vaccination program. This study also included 100 serum samples from healthy adult blood bank donors (HEMORIO, Rio de Janeiro, Brazil). This study was approved by the UNIGRANRIO study center ethics committee (CAAE: 24856610.0.0000.5283) and was conducted in accordance with good clinical practice and all applicable regulatory requirements, including the Declaration of Helsinki.

### 2.3. Synthesis of the Cellulose-Membrane-Bound Peptide Array and Screening

Two libraries of 192 amino acids overlapping fourteen peptides, shifted by 5 residues, covering the primary sequence of S1 (P04977.1), S2 (P04978.2), S3 (P04979.1), S4 (P0A3R5.1), S5 (P04981.5), Ptx were synthesized directly onto amino-PEG500-UC540 cellulose membranes. This was achieved using the SPOT synthesis technique, as previously described [28], with an Auto-Spot Robot ASP-222 (Intavis Bioanalytical Instruments AG, Köln, Germany) and the F-moc strategy. For the immunodetection assays, the membranes were washed with TBS (50 mM Tris-buffer saline, pH 7.0) and blocked overnight with TBS-CT (Tris-buffer saline, 3% casein, 0.1% Tween 20, pH 7.0) at room temperature under agitation overnight at 4 °C. After extensive washing with TBS-T (Tris-buffer saline, 0.1% Tween 20, pH 7.0) to remove any unbound or non-specifically bound peptides the membranes presenting the peptide libraries were incubated for 2 h with a pool of mice (miVs) or children vaccinated sera (chVs, 1:100) in TBS-CT and then washed again with TBS-T.

After new steps of washing with PBS-T, membranes were incubated for 1 h with goat anti-mouse IgG (KPL, Gaithersburg, MD, USA) or goat anti-human IgG (Sigma-Aldrich, St Louis, MO, USA) conjugated to alkaline phosphatase (1:5000) for 1 h at 37 °C, followed by washing with TBS-T and CBS (50 mM citrate-buffer saline, pH 7.0). To complete the reaction, chemiluminescent CDP-Star^®^ Substrate (0.25 mM) with Nitro-Block-II™ Enhancer (Applied Biosystems, Waltham, MA, USA) was added.

Chemiluminescent signals were scanned and measured using an Odyssey FC (LI-COR Bioscience, Lincoln, NE, USA), as previously described [29]. In brief, a 5 MP digital image file was generated, and signal intensities were quantified using TotalLab TL100 (v. 2009; Nonlinear Dynamics, Newcastle upon Tyne, UK) software. The signal intensity (SI) used as a background was determined by a set of negative controls spotted on each membrane. Negative controls (without peptide or with a non-antigenic peptide from *Clostridium tetani* (IHLVNNESSEVIVHK) and positive controls (GYPKDGNAFNNLD RI-*Clostridium tetani*–A10 and KEVPALTAVE TGATN–Poliovirus–A11) were included.

### 2.4. Bioinformatics Analysis and In Silico Models

The complete protein sequences of interest [subunits S1 (P04977.1), S2 (P04978.2), S3 (P04979.1), S4 (P0A3R5.1), S5 (P04981.5)] of the Ptx were initially retrieved from the National Center for Biotechnology Information, USA (http://www.ncbi.nlm.nih.gov, accessed 10 September 2025).

To identify epitope locations within the 3D structures of these proteins, we generated models using UCSF ChimeraX, Version 1.11 (https://www.rbvi.ucsf.edu/chimerax/; accessed on 20 September 2025). The structures of the five proteins were generated using the AlphaFold server [30] and the Protein Data Bank (PDB). Searches for pertussis toxin subunits were conducted in the Protein Information Resource (PIR) database (https://research.bioinformatics.udel.edu/peptidematch/index.jsp, accessed on 10 November 2025) using previously identified sequences in other organisms.

Similarity analysis with proteins from other organisms was conducted using BLASTP version 4.0. Multiple sequence alignments were performed using the programs ClustalW (https://www.ebi.ac.uk/jdispatcher/msa/clustalo (accessed on 12 September 2025) and BioEdit (https://bioedit.software.informer.com/7.2/ (accessed on 12 September 2025).

### 2.5. Structural Modeling of Pertussis Toxin and Epitope Mapping

The three-dimensional structure of the Ptx-S1 was obtained from experimentally resolved structural data deposited in the PDB. Structural analyses were conducted using PyMOL (Schrödinger LLC, New York, NY, USA) and UCSF ChimeraX to delineate the architecture of the enzymatically active center responsible for NAD^+^-dependent ADP-ribosyltransferase activity.

### 2.6. Antigen–Antibody Interface

Structural complexes of *Bordetella pertussis* toxin bound to neutralizing antibodies were identified by searching the Protein Data Bank (PDB). A total of five unique antibody–antigen complex structures were retrieved and analyzed: 9MR7, 9E3L, 9E3H, 9E3J, and 9E3K. Duplicate entries were excluded from subsequent analyses.

The selected structures were used to investigate the binding mode of antibody fragments (Fabs) to Ptx subunits and to characterize interactions involving previously identified or putative epitopes. Structural visualization, inspection, and comparative analyses were performed using UCSF ChimeraX (version 1.11) and PyMOL (Schrödinger, version 4.6.0). These tools were employed to examine antibody orientation, paratope–epitope complementarity, and the contribution of individual complementarity-determining regions (CDRs) to antigen recognition.

A refined and systematic analysis of non-covalent interactions at the antigen–antibody interface was conducted using the Protein–Ligand Interaction Profiler (PLIP) web server. PLIP was used to identify and classify intermolecular interactions, including hydrogen bonds, electrostatic (salt-bridge) interactions, hydrophobic contacts, π–π stacking, and cation–π interactions, based on side-chain geometry and distance criteria. Interaction profiles were generated for each complex and compared to identify conserved binding features and structural determinants associated with antibody neutralization.

## 3. Results

### 3.1. Epitope Mapping

**Figure 1:**
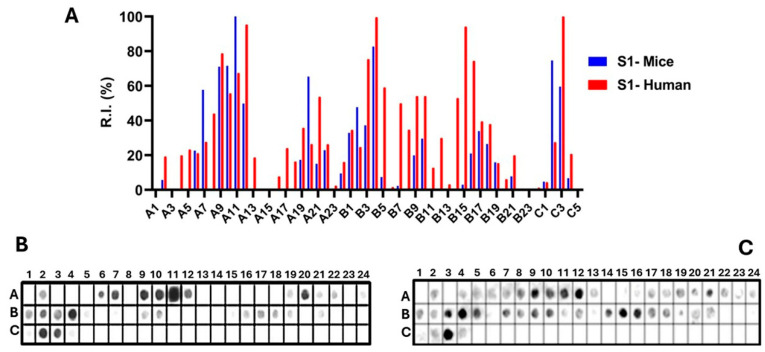
Epitope mapping of PtxS1. (**A**) Quantitative analysis of chemiluminescent signal intensities obtained from peptide array membranes incubated with pooled sera from miVs (blue) and chVs (red). The *y*-axis represents relative signal intensity (R.I. %), and the *x*-axis (A1–C5) indicates the corresponding synthetic peptides (see Table S1). (**B**,**C**) Representative images of peptide array membranes synthesized in parallel and incubated with miVs (**B**) and chVs (**C**), respectively. Each spot corresponds to a 14-amino-acid peptide with a 9-residue overlap, collectively covering the full PtxS1 sequence.

**Figure 2:**
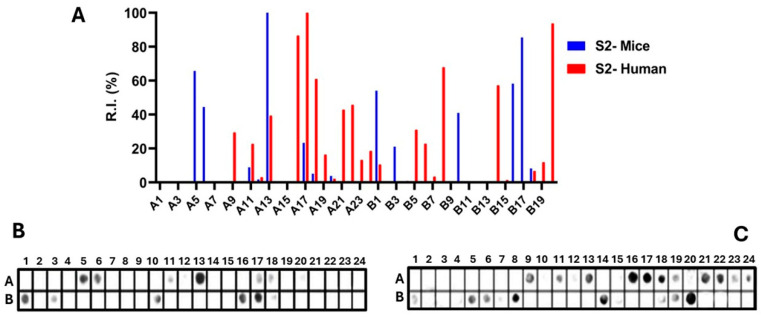
Epitope mapping of the PtxS2. (**A**) Quantitative analysis of chemiluminescent signal intensities obtained from peptide array membranes incubated with pooled sera from miVs (blue) and chVs (red). The *y*-axis represents relative signal intensity (R.I. %), and the *x*-axis (A1–B20) indicates the corresponding synthetic peptides (see Table S1). (**B**,**C**) Representative images of peptide array membranes synthesized in parallel and incubated with miVs (**B**) and chVs (**C**), respectively. Each spot corresponds to a 14-amino-acid peptide with a 9-residue overlap, collectively covering the full PtxS1 sequence.

**Figure 3:**
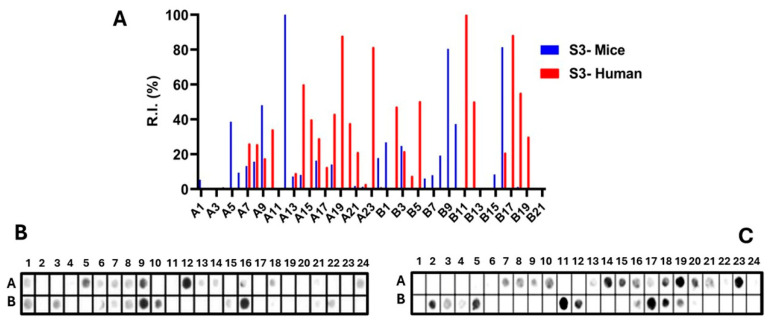
Epitope mapping of the PtxS3. (**A**) Quantitative analysis of chemiluminescent signal intensities obtained from peptide array membranes incubated with pooled sera from miVs (blue) and chVs (red). The *y*-axis represents relative signal intensity (R.I. %), and the *x*-axis (A1–B21) indicates the corresponding synthetic peptides (see Table S1). (**B**,**C**) Representative images of peptide array membranes synthesized in parallel and incubated with miVs (**B**) and chVs (**C**), respectively. Each spot corresponds to a 14-amino-acid peptide with a 9-residue overlap, collectively covering the full PtxS1 sequence.

**Figure 4:**
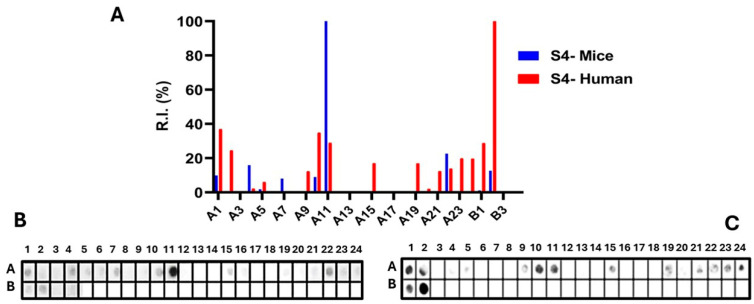
Epitope mapping of the PtxS4. (**A**) Quantitative analysis of chemiluminescent signal intensities obtained from peptide array membranes incubated with pooled sera from miVs (blue) and chVs (red). The *y*-axis represents relative signal intensity (R.I. %), and the *x*-axis (A1–B3) indicates the corresponding synthetic peptides (see Table S1). (**B**,**C**) Representative images of peptide array membranes synthesized in parallel and incubated with miVs (**B**) and chVs (**C**), respectively. Each spot corresponds to a 14-amino-acid peptide with a 9-residue overlap, collectively covering the full PtxS1 sequence.

**Figure 5:**
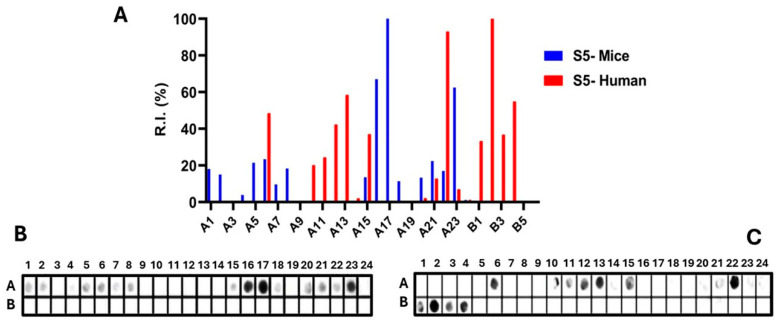
Epitope mapping of the PtxS5. (**A**) Quantitative analysis of chemiluminescent signal intensities obtained from peptide array membranes incubated with pooled sera from miVs (blue) and chVs (red). The *y*-axis represents relative signal intensity (R.I. %), and the *x*-axis (A1–B5) indicates the corresponding synthetic peptides (see Table S1). (**B**, **C**) Representative images of peptide array membranes synthesized in parallel and incubated with miVs (**B**) and chVs (**C**), respectively. Each spot corresponds to a 14-amino-acid peptide with a 9-residue overlap, collectively covering the full PtxS1 sequence.

Epitopes in the Ptx subunit protein (total of 960 residues) were identified by recognizing peptides in synthesized libraries by children’s and murine antibodies immunized with a wPv. Figure 1, Figure 2, Figure 3, Figure 4 and Figure 5, Panels B and C present the position of each peptide derived from each subunit (PtxS1, PtxS2, PtxS3, PtxS4, and Ptx5) and the measured intensity (panels A) from the chemiluminescent detection of mice and children’s IgG antibodies in sera pooled from immunized children with the wPv. The intensities were normalized to 100% using the positive control.

A list of the Ptx subunit peptides sequences synthesized and their positions on the membranes is presented in Figure S1. The pattern of reactivity of antibodies generated in children immunized with the wPv showed that more peptides were recognized than in mice immunized with the wPv vaccine (Table 1).

An analysis of peptide sequences synthesized in reactive regions identified 20 epitopes recognized by miVs and 24 by chVs (Table 1). Eight epitopes were uniquely specified by miVs (Ep2,11,14,16,17,19,29,31) and thirteen by the chVs (Ep6,8,12,13,15,18,22–24,27,29,30,31). The remaining epitopes were common between the two species (Table 1). Overall, the epitopes were designated as Ep1–Ep32 for this study.

### 3.2. Localization of the B-Epitopes Within the Ptx Protein

A total of 32 linear B-epitopes were identified by SPOT-synthesis analysis in the sera of children and/or immunized mice. The Ptx protein gene contains five well-defined chains: S1 (269 residues), S2 (226 residues), S3 (227 residues), S4 (227 residues), and S5 (133 residues). Nine epitopes (Ep1, Ep3–10) were identified by the chVs and nine (Ep1–5 and Ep7, Ep9 and Ep10) by miVs in the PtxS1 chain. Four epitopes (Ep12, Ep13, Ep15, Ep18) were detected by chVs and four (Ep11, Ep14, Ep16 and Ep17) in the PtxS2. Six (Ep20–Ep26) were detected by chVs, and four (Ep19–Ep21, Ep25, and Ep26) by miVs in PtxS3. In S4, only one epitope was reactive to miVs (Ep28), and three epitopes (Ep27, Ep29, Ep30) were identified exclusively for chVs (Table 1). Two epitopes were found in S5 for miVs (Ep31 and 32), and Ep32 was shared by chVs.

The shared epitopes in the PtxS1 were six (Ep1, Ep3, Ep4, Ep5, Ep7, Ep9 and Ep10), in the PtxS2 (none), in the PtxS3 (four; Ep20, Ep21, Ep25 and Ep26), PtxS4 (none), and in the PtxS5 (one; Ep32) (Table 1).

### 3.3. Spatial Location of the Ptx Reactive Epitopes

**Figure 6:**
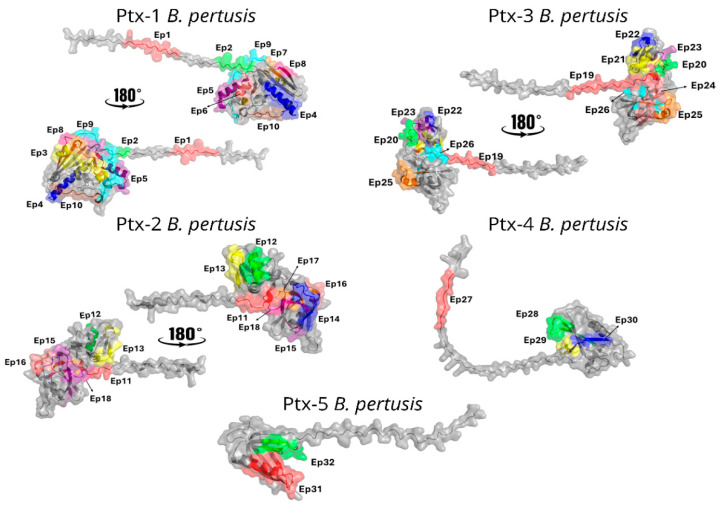
Spatial distribution of IgG reactive epitopes. The figure depicts the spatial positioning of children- and mouse-IgG-reactive epitopes (Table 1) within the S1–S5 domain of the Ptx.

The tridimensional structures of mature Ptx have been determined to a resolution of 2.5 Å by X-ray diffraction [34,35]. In our study, a predicted structural model of the S1–S5 subunits was obtained (Figure 6) using AlphaFold and displays the spatial localization of the most reactive epitopes identified by the SPOT-synthesis array experiments. Most of the linear epitopes were in coil/loop structures in the S1–S5 protein structure. The hydropathy plots of the Ptx subunits also suggested that all epitopes were on the protein surface.

### 3.4. Distribution of Antibody Binding Across Ptx and Epitope Hotspots

Analysis of the available pertussis toxin–antibody complex structures revealed a non-uniform distribution of antibody binding across toxin subunits. Antibody interactions were predominantly observed with subunit S3, which accounted for the most binding events (*n* = 16), indicating that this subunit represents a major immunogenic and structurally accessible region of the toxin. In contrast, subunits S2 and S4 showed a moderate number of antibody interactions (*n* = 7 each), suggesting secondary but relevant roles in antibody recognition. Only a single antibody interaction was detected for subunit S1 (*n* = 1), indicating limited structural targeting of this subunit by neutralizing antibodies within the currently available structural dataset. Interestingly, S1/Ep5 represents the catalytic domain, which was located within the core of the catalytic cleft (residues 136–149), whereas Ep3 (residues 63–70) and Ep4 (residues 105–113, NAD^+^ site) occupied adjacent loops. The Ep3 is located between Ep4 and Ep5 and is a secondary structural element that frames the active site. Together, these regions define an extended functional epitope spanning the toxin’s enzymatic center.

**Figure 7:**
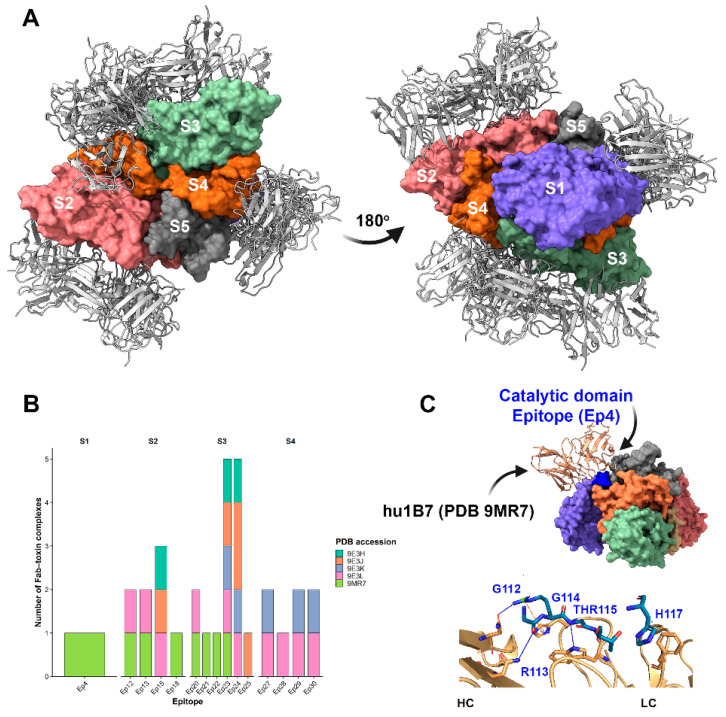
Structural and quantitative analysis of pertussis toxin–antibody interactions. (**A**) Structural overview of Ptx in complex with neutralizing antibody Fab fragments derived from five deposited crystal or cryo-EM structures (PDB IDs: 9MR7, 9E3L, 9E3H, 9E3J, and 9E3K). The toxin subunits (S1–S5) are shown as surface or cartoon representations, with bound Fab fragments displayed to highlight differences in antibody orientation and subunit specificity across complexes. (**B**) Quantitative distribution of antibody binding across Ptx subunits. The number of antibodies interacting with each subunit (S1–S5) was determined from structural analyses, revealing a predominant targeting of the S3 subunit, followed by S2 and S4, with limited binding to the catalytic S1 subunit (Ep4, aa 105–113). (**C**) Detailed interaction map of the monoclonal antibody hu1B7 bound to the S1 subunit at epitope Ep4 (PDB ID: 9MR7). The antibody light chain (LC) and heavy chain (HC) are shown engaging the catalytic subunit through a network of non-covalent interactions. Hydrogen bonds are depicted as blue dashed lines, while π–cation interactions are shown as yellow dashed lines, illustrating the molecular basis of antibody recognition and the potential interference with the toxin’s enzymatic activity.

At the epitope level, interaction mapping identified distinct epitope hotspots that were repeatedly targeted by multiple antibodies. Epitopes Ep23 and Ep24 emerged as the most frequently recognized regions, each interacting with five independent antibodies, highlighting their potential role as dominant antigenic sites. Epitope Ep15 showed intermediate recognition (*n* = 3), while a group of epitopes (Ep12, Ep13, Ep20, Ep27, Ep29, and Ep30) displayed moderate antibody engagement (*n* = 2 each). The remaining epitopes (Ep18, Ep21, Ep22, Ep25, Ep28, and Ep4) were each recognized by a single antibody, suggesting more restricted or context-dependent accessibility (Figure 7).

### 3.5. Structural Basis of Toxin Neutralization

Antibody-mediated neutralization of pertussis toxin can occur through multiple mechanisms, including steric hindrance of receptor binding, destabilization of toxin assembly, or direct interference with the catalytic activity of the enzymatic subunit. Although most antibody interactions identified in this study were concentrated on the S2, S3, and S4 subunits, a limited but noteworthy interaction with the S1 subunit, which harbors the catalytic domain, was observed. Given the central role of S1 in ADP-ribosyltransferase activity, antibodies targeting this subunit may directly inhibit toxin function.

To explore this possibility, we performed a detailed structural analysis of PDB entry 9MR7, which represents the monoclonal antibody hu1B7 bound to the Ep4 epitope on the S1 subunit. This antibody has been reported to recognize both human and murine pertussis toxin [35], making it particularly relevant for functional interpretation. Structural inspection revealed that hu1B7 engages the catalytic subunit via a network of specific noncovalent interactions between the light and heavy chains.

The light chain contributes to antigen recognition through a combination of hydrophobic contacts, hydrogen bonds, and π–cation interactions. A hydrophobic interaction involving residue HIS117A was detected at a distance of 3.65 Å, suggesting stabilization of the antibody–toxin interface. In addition, hydrogen bonds formed by GLY114A (2.56 Å) and THR115A (2.99 Å) further anchor the antibody to the epitope. A π–cation interaction involving ARG113A at 4.74 Å likely reinforces binding specificity and orientation.

The heavy chain establishes additional stabilizing contacts through hydrogen bonds involving GLY112A (3.18 Å) and ARG113A (1.92 Å). Notably, the short interaction distance observed for ARG113A indicates strong, potentially functionally relevant contact that may constrain the local conformational flexibility of the S1 subunit.

Importantly, the localization of Ep4 within the S1 subunit places these interactions near the toxin’s catalytic region, suggesting a neutralization mechanism based on direct steric obstruction or conformational restriction of the enzymatic domain (Figure 7). Such binding may impair substrate access or disrupt the proper positioning of residues required for ADP-ribosyltransferase activity. Together, these findings support a model in which a subset of neutralizing antibodies directly targets the catalytic subunit, providing an additional, mechanistically distinct layer of toxin neutralization.

### 3.6. Cross-Immunity Conferred with Other Strains of B. pertussis

To investigate the cross-immunity conferred by the PtxS1-S5 subunits of *B. pertussis* and *B. parapertussis,* and by other adhesin proteins from other organisms, a set of sequences deposited in the Uniprot database was aligned to compare epitope sequences.

This analysis showed that, in total, fourteen epitopes were unique to *B pertussis* (Ep1, Ep2, Ep3, Ep17, E18, Ep23, Ep25–Ep32). The S1 presented three (Ep1–Ep3), the S2 two (Ep17 and Ep18), the S3 two (Ep25 and Ep26), and all the epitopes from S4 and all the S5 (Ep31–Ep32) (Table S1). The high conservation of epitope structure suggests that strong cross-immunity occurs upon immunization with *B. pertussis* and *B. parapertussis* toxin preparations.

### 3.7. Search for Similar Epitope Sequences in the Data Bank

A literature search showed that some Ptx epitopes had previously been identified using monoclonal antibodies or rabbit-immunized sera (Table 1). Our epitope mapping revealed thirteen (S1-6, S2-2, S3-2, S4-1 and S5-2) new murine and nineteen (S1-6, S2-3, S3-5, S4-3 and S5-1) new human epitopes.

The epitopes recognized by chVs [S1 (Ep6,8); S2 (Ep12, Ep13, Ep15, Ep18), S3 (Ep22–Ep24); S4 (Ep27, Ep29, Ep30)] were unique based on sequence homology in the database. Four of these distinct epitopes were identified in the S2 subunit for each species [mice Ep11, 14, 16, 17 and children Ep12, 13, 15, 18], and only one in the S4 subunit (murine Ep28).

### 3.8. Correlation of Ptx Within Bordetella sp.

Since adhesin structures are highly conserved, proteins in this class are expected to exhibit structural similarity. As shown in Figure S2, the PtxS1–S5 proteins each harbor a set of linear epitopes that are also present in other Bordetella adhesin proteins.

Our study identified 11 *B. pertussis* epitopes that share sequence similarity with the *B. parapertussis* toxin, including the catalytic center (aa 136–149), the NAD^+^ binding block (aa 105–113), and the possible anchoring epitope site (aa 63–70) (Figure S2). However, these epitopes were recognized differently by sera from immunized mice and children (Table 1). The epitopes recognized by chVs that share sequences with *B. parapertussis* are Ep4–7, Ep12, Ep13, Ep15, Ep16, Ep20–24, and those recognized by miVs are Ep4, Ep5, Ep7, Ep9–11, Ep14, Ep16, Ep19–21 (Table 1).

However, a set of nine epitopes (Ep1, Ep3, Ep18, Ep25, Ep26, Ep28, and Ep30–32) from *B. pertussis* recognized by chVs does not share sequence similarity with the toxin of *B. parapertussis.*

## 4. Discussion

In this study, we performed a comprehensive linear B-cell epitope mapping of the five subunits of pertussis toxin (PtxS1–S5) using sera from whole-cell pertussis–vaccinated mice and children. By combining SPOT-synthesis peptide arrays, quantitative chemiluminescent detection, and structural modeling, we identified 32 IgG-reactive linear epitopes and demonstrated marked qualitative and quantitative differences between murine and human antibody repertoires. These findings provide mechanistic insight into interspecies differences in pertussis immunity and have direct implications for vaccine design, diagnostics, and translational modeling.

Pertussis toxin comprises five subunits (S1–S5) organized into two functional components: the toxic A subunit (S1) and the B subunit, which mediates receptor binding and consists of S2, S3, S5, and two S4 molecules. The assembly is stabilized by S5, which connects the S2–S4 and S3–S4 dimers. Consistent with previous reports, murine immunization with whole-cell pertussis vaccines elicited a focused antibody response that preferentially targets conserved, surface-exposed linear regions of the B-oligomer subunits S2, S3, and S4 [21,31,36,37]. The Ep3 sequence recognized by murine antibodies does not appear to fully block PtxS1 enzymatic activity, as only two residues (63 and 64) in site 1 of the active center were identified (Table 1). However, it is possible that the murine antibody binding to a site adjacent to the active center blocks substrate access or causes steric hindrance. This is consistent with previous findings that several monoclonal antibodies may block ADP ribosylation in vitro but are not neutralizing in vivo [38].

In fact, the predominance of murine epitopes in these subunits also aligns with earlier studies showing that mice tend to recognize linear peptide determinants on the toxin surface that are associated with receptor binding rather than enzymatic activity [7,39]. This pattern likely reflects species-specific constraints on the B-cell repertoire and antigen-processing pathways, as well as the Th1 (IFN-¥)/Th17 (IL-17)-biased immune milieu induced by whole-cell vaccination in mice [40,41].

In contrast, sera from vaccinated children recognized a broader array of linear epitopes across all five Ptx subunits, with a clear enrichment in the S1 enzymatic domain (essential for toxicity) and in regions proximal to known receptor-binding interfaces (adhesion) in S2 and S3 [22,30,32]. This broader recognition profile is consistent with prior observations that human immune responses to Ptx frequently include antibodies directed against enzymatically active and functionally neutralizing regions of the toxin [41,42,43]. Notably, thirteen epitopes were uniquely identified by children’s sera, many of which have not been previously reported (Table 1 and Table S1), underscoring the greater diversity and complexity of the human antibody response to Ptx.

The limited overlap between murine and human epitope repertoires further highlights the challenges of extrapolating antibody specificity data from animal models to human immunity. Only a subset of epitopes, particularly within PtxS1, PtxS3, and PtxS5, was commonly recognized by both species. This partial convergence suggests that while mice remain a valuable model for identifying conserved and immunogenic linear determinants, they may underrepresent epitopes associated with toxin neutralization and long-term protection in humans [41,42]. These findings reinforce prior concerns about the translational limitations of murine pertussis models when used in isolation [41,43].

Structural mapping of the identified epitopes revealed that most localized to loop and coil regions exposed on the surface of the mature Ptx holotoxin, consistent with crystallographic data and hydropathy predictions [44,45]. The surface accessibility of these epitopes supports their biological relevance and explains their immunodominance following vaccination. Importantly, several human-specific epitopes mapped to regions involved in enzymatic activity or receptor engagement [39,46,47], providing a plausible mechanistic basis for the higher neutralizing capacity often observed in human anti-Ptx antibodies.

The structural patterns observed in this study are highly consistent with the mechanistic framework recently described using cryo-EM, which identified that a neutralizing antibody binds to Ptx [35]. The structural analysis of the two humanized monoclonal antibodies studied, hu11E6 and hu1B7, that target distinct functional epitopes on genetically detoxified PT (PTg), with hu11E6 binding a conserved epitope on the paralogous S2 and S3 subunits to block toxin adhesion to sialylated receptors, thereby preventing cell binding and mitogenic activity, whereas hu1B7 engages a epitope spanning the S1 and S5 subunits and neutralizes PT through interference with its intracellular activity rather than through S5 contact itself [35]. Our quantitative and structural analyses corroborate this model by revealing a dominant antibody-binding bias toward the S3 subunit, followed by S2 and S4, consistent with the preferential targeting of structurally exposed and immunodominant regions. The recurrence of antibody engagement at specific epitope hotspots (notably Ep23 and Ep24) further supports the concept of conserved antigenic surfaces that accommodate diverse neutralizing antibodies.

Importantly, the identification of Ep4-binding hu1B7 on the S1 subunit extends previous observations [35] by providing additional structural evidence for direct targeting of the catalytic domain as a neutralization strategy.

These structural findings have direct implications for the design of acellular pertussis vaccines. As highlighted in Figure 7, epitopes proximal to/or overlapping the catalytic center, particularly Ep3, Ep4, and Ep5, represent high-value targets for structure-guided immunogen development. Incorporating or enhancing such epitopes in vaccine antigens may preferentially elicit antibodies with true neutralizing capacity rather than antibodies that bind without functional inhibition. This approach aligns with emerging strategies to improve the effectiveness of acellular pertussis vaccines by focusing immune responses on mechanistically relevant regions of the toxin.

Overall, integrating structural modeling with epitope-level immunological data supports a mechanism-driven framework for vaccine optimization, in which antibody responses are directed toward active-site-associated epitopes that directly impair toxin function.

In addition, the analysis of epitope conservation across Bordetella species demonstrated that while many epitopes are shared between *B. pertussis* and *B. parapertussis*, a distinct subset, particularly within PtxS1 and PtxS5, was unique to *B. pertussis*. These species-specific epitopes, predominantly recognized by children’s sera, may contribute to differential cross-protection and could be exploited to improve diagnostic specificity or to develop next-generation vaccine formulations [35]. Conversely, the high conservation of several epitopes supports the concept of partial cross-immunity between *Bordetella* species following whole-cell vaccination, as previously suggested [48,49,50].

Our findings also expand the current epitope landscape of Ptx. Comparison with previously described monoclonal antibody and rabbit antisera studies identified 11 novel murine and 16 novel human linear epitopes. This expanded repertoire provides a valuable resource for rational antigen selection, particularly for epitope-based vaccines and serological assays that distinguish vaccine-induced from infection-induced immunity.

## 5. Conclusions

This study provides a comprehensive linear B-cell epitope map of pertussis toxin across all five subunits, revealing pronounced qualitative differences in antibody responses between murine and human vaccination. While mice predominantly targeted conserved, surface-exposed epitopes within the B-oligomer subunits S2, S3, and S4, vaccinated children exhibited broader epitope recognition, with marked enrichment in the S1 enzymatic domain and functionally relevant regions of the toxin.

These findings provide a mechanistic basis for differences in toxin neutralization and highlight limitations in extrapolating murine epitope data to human immunity.

Together, our results support a structure-guided approach to pertussis vaccine design that prioritizes active-site–associated epitopes with demonstrated neutralizing capacity and expands the epitope framework available for improved diagnostics and translational modeling.

## Figures and Tables

**Table 1:** Relationship of Ptx IgG epitopes identified by sera from cellular pertussis vaccinated mice (miVs) and children (chVs).

Epitope Number	Ptx-S1 Mice	S1-Children	Cross Reactivity	References
**Ep1**	**^11^** **ARTGWLTWLA^20^**	**^11^** **ARTGWLTWLA^20^**		**aa 3–16/mAb** [31] and Rab* [32]
**Ep2**	**^31^** **PAWADDPPA^39^**			
**Ep3**	**^46^** **SRPPEDVFQNGFTAWGNND^64^**	**^61^** **GNND** **NVLDHL^70^**		
**Ep4**	**^96^** **VYLEHRMQEAVEAE^109^**	**^105^** **AVEAE** **RAGRGTGH^117^**	**BPp**	**PDB: 9MR7** [33] **(huAb)**
**Ep5**	**^126^** **RADNNFYGAASSYFEYVDT^144^**	**^136^** **SSYFE YVDTYGDNA^149^**	**BPp**	aa 121–138/Rab* [31]
**Ep6**		**^156^** **ALATYQSEY^164^**	**BPp**	
**Ep7**	**^166^** **AHRRIPPEN^174^**	**^170^** **IPPEN** **IRRVT^179^**	**BPp**	
**Ep8**		**^191^** **TTTEYSNAR^199^**	**BPp**	
**Ep9**	**^201^** **VSQQTRANPNPYTSRRSVA^219^**	**^211^** **PYTSRRSVA** **SIVGT^224^**	**BPp**	aa 211–222/Rab* [32] **aa 201–235/mice** [31]
**Ep10**	**^251^** **ERAGEAMVL** **^259^**	**^251^** **ERAGEAMVL** **VYYES^264^**	**BPp**	
	**S2 mice**	**S2-children**		
**Ep11**	**^26^** **RASTPGIVI^34^**		**BPp**	aa 1–23/Rab* [33]
**Ep12**		**^66^** **GDLQEYLRH^74^**	**BPp**	**PDB: 9MR7** [22] **and PDB: 9E3L(huAb)**
**Ep13**		**^91^** **GGEYGGVIKDGTPG^104^**	**BPp**	**PDB: 9MR7** [22] **and PDB: 9E3L(huAb)**
**Ep14**	**^121^** **TGQPATDHY^129^**		**BPp**	
**Ep15**		**^131^** **SNVTATRLLS STNS^144^**	**BPp**	aa134–149/Rab* [31] **and** **PDB: 9E3L/9E3H/9E3J (huAb)**
**Ep16**	**^161^** **CTSPYDGKYWSMYS^174^**		**BPp**	
**Ep17**	**^195^** **SKEEQYYD^202^**			aa 186–199/Rab* [31]
**Ep18**		**^205^** **DATFETYALT^214^**		**PDB: 9MR7** [22] **(huAb)**
	**S3-mice**	**S3-children**		
**Ep19**	**^21^** **LGMRTAQAVAPGIVIPPKAL^34^**		**BPp**	aa 18–41/Rab* [33]
**Ep20**	**^41^** **FTQQ** **GGAYGRC^51^**	**^31^** **PGIVIPPKALFTQQ^44^**	**BPp**	aa 37–64/Rab* [33] **and PDB: 9MR7** [22]**/9E3L(huAb)**
**Ep21**	**^56^** **RALTVAELRGNAEL^69^**	**^66^** **NAEL** **QTYLR^74^**	**BPp**	**PDB: 9MR7** [22] **(huAb)**
**Ep22**		**^86^** **YDGTYGQAYGGII^99^**	**BPp**	**PDB: 9MR7** [22] **(huAb)**
**Ep23**		**^106^** **AGFIYRETF^114^**	**BPp**	**PDB: 9MR7** [22]**/9E3L/9E3K/9E3H/9E3J(huAb)**
**Ep24**		**^116^** **ITTIYKTGQPAADH^1^**	**BPp**	**PDB: 9E3L/9E3K/9E3H/9E3J(huAb)**
**Ep25**	**^161^** **ACASPYEGRYRDMY^174^**	**^169^** **YRDMY** **DALRR^179^**		aa 149–176/Rab* [33]**/PDB: 9E3J(huAb)**
**Ep26**	**^196^** **SKEEQYYDYED^206^**	**^200^** **QYYDYEDATF^209^**		aa 18–41/Rab* [33]
	**S4-mice**	**S4-children**		
**Ep27**		**^06^** **PTRTTAPGQ^14^**		**PDB: 9E3L/9E3K(huAb)**
**Ep28**	**^56^** **TSVAMKPYEVTPTR^69^**			**PDB: 9E3L(huAb)**
**Ep29**		**^125^** **GPKQLTFEGK^134^**		**PDB: 9E3L/9E3K(huAb)**
**Ep30**		**^136^** **ALELIRMV^143^**		**PDB: 9E3L/9E3K (huAb)**
	**S5-mice**	**S5-children**		
**Ep31**	**^76^** **LSDAGHEHDTWFDTMLGFA^9^**			
**Ep32**	**^111^** **SPYP** **GTPGDLLEL^123^**	**^106^** **LTVEDSPYP^114^**		

* Rabbit antisera raised against synthetic peptides; BPp, *Bordetella parapertussis*; mAb, monoclonal antibodies. The common epitope residues recognized by both species are shown in gray. PDB, protein data bank (https://www.rcsb.org/?ref=nav_home, Accessed on 12 December 2025).

## Data Availability

The data presented in this study are available on request from the corresponding author.

## Supplementary Materials

The following supporting information can be downloaded at: https://www.mdpi.com/article/10.3390/vaccines14050413/s1, Figure S1. List of overlapping 14-mer peptides derived from all subunits of *Bordetella pertussis* toxin (Ptx) and analyzed by SPOT-synthesis. Peptides were designed to comprehensively cover the full-length sequences of each toxin subunit, with sequential overlap to ensure continuous epitope mapping. Each peptide corresponds to a defined position within its respective subunit and was synthesized on cellulose support for antibody-binding analysis. This approach enabled the systematic identification of linear B-cell epitopes across the entire pertussis toxin complex. Figure S2. Homology analysis of pertussis and parapertussis toxins, demonstrating the similarity of the epitopes detected in the Ptx(s) subunits. For toxins subunit 4 and 5, *B. pertussis* lacks a toxin homologous to that of *B. parapertussis*, and no other human pathogen harbors a toxin with substantial similarity. Table S1. UniProt-reviewed bacterial toxins and homologous adhesins were analyzed for potential epitope cross-reactivity. The dataset comprises the following UniProt entries: P01555, P01556, P43530, P32890, P08191, P13430, P43261, Q5HD51, Q53653, and Q53654. The IEDB epitope similarity analysis shows the percentage of protein sequences that match known epitopes at ≤50% identity.
