# Supplementary material for: Screening Epitopes Through Comparative Analysis of Children and Mice Immune Responses to Pertussis Toxin Subunits (S1–S5) Induced by Whole-Cell Pertussis Vaccination
Source: Vaccines (Basel). 2026 May 2;14(5):413. doi: 10.3390/vaccines14050413 (PMC13211744; doi:10.3390/vaccines14050413)
Supplement: Supplementary file 1 [file vaccines-14-00413-s001.zip › vaccines-4181699-supplementary.pdf]

	MRCTRAIRQTARTG
A 2	AIRQTARTGWLTWL
A 3	ARTGWLTWLAILAV
A 4	LTWLAILAVTAPVT
A 5	ILAVTAPVTSPAWA
A 6	APVTSPAWADDPPA
A 7	PAWADDPPATVYRY
A 8	DPPATVYRYDSRPP
A 9	VYRYDSRPPEDVFQ
A10	SRPPEDVFQNGFTA
A11	DVFQNGFTAWGNND
A12	GFTAWGNNDNVLDH
A13	GNNDNVLDHLTGRS
A14	VLDHLTGRSCQVGS
A15	TGRSCQVGSSNSAF
A16	QVGSSNSAFVSTSS
A17	NSAFVSTSSSRRYT
A18	STSSSRRYTEVYLE
A19	RRYTEVYLEHRMQE
A20	VYLEHRMQEAVEAE
A21	RMQEAVEAERAGRG
A22	VEAERAGRGTGHFI
A23	AGRGTGHFIGYIYE
A24	GHFIGYIYEVRADN
B 1	YIYEVRADNNFYGA
B 2	RADNNFYGAASSYF
B 3	FYGAASSYFEYVDT
B 4	SSYFEYVDTYGDNA
B 5	YVDTYGDNAGRILA
B 6	GDNAGRILAGALAT
B 7	RILAGALATYQSEY
B 8	ALATYQSEYLAHRR
B 9	QSEYLAHRRIPPEN
B10	AHRRIPPENIRRVT
B11	PPENIRRVTRVYHN
B12	RRVTRVYHNGITGE
B13	VYHNGITGETTTTE
B14	ITGETTTTEYSNAR
B15	TTTEYSNARYVSQQ
B16	SNARYVSQQTRANP
B17	VSQQTRANPNPYTS
B18	RANPNPYTSRRSVA
B19	PYTSRRSVASIVGT
B20	RSVASIVGTLVRMA
B21	IVGTLVRMAPVIGA
B22	VRMAPVIGACMARQ
B23	VIGACMARQAESSE
B24	MARQAESSEAMAAW
C 1	ESSEAMAAWSERAG
C 2	MAAWSERAGEAMVL

C 3	ERAGEAMVLVYYES
C 4	AMVLVYYESIAYSF
C 5	
C 6	MPIDRKTLCHLLSV
C 7	KTLCHLLSVLPLAL
C 8	LLSVLPLALLGSHV
C 9	PLALLGSHVARAST
C10	GSHVARASTPGIVI
C11	RASTPGIVIPPQEQ
C12	GIVIPPQEQITQHG
C13	PQEQITQHGGPYGR
C14	TQHGGPYGRCANKT
C15	PYGRCANKTRALTV
C16	ANKTRALTVAELRG
C17	ALTVAELRGSGDLQ
C18	ELRGSGDLQEYLRH
C19	GDLQEYLRHVTRGW
C20	YLRHVTRGWSIFAL
C21	TRGWSIFALYDGTY
C22	IFALYDGTYLGGEY
C23	DGTYLGGEYGGVIK
C24	GGEYGGVIKDGTPG
D 1	GVIKDGTPGGAFDL
D 2	GTPGGAFDLKTTFC
D 3	AFDLKTTFCIMTTR
D 4	TTFCIMTTRNTGQP
D 5	MTTRNTGQPATDHY
D 6	TGQPATDHYYSNVT
D 7	TDHYYSNVTATRLL
D 8	SNVTATRLLSSTNS
D 9	TRLLSSTNSRLCAV
D10	STNSRLCAVFVRSG
D11	LCAVFVRSGQPVIG
D12	VRSGQPVIGACTSP
D13	PVIGACTSPYDGKY
D14	CTSPYDGKYWSMYS
D15	DGKYWSMYSRLRKM
D16	SMYSRLRKMLYLIY
D17	LRKMLYLIYVAGIS
D18	YLIYVAGISVRVHV
D19	AGISVRVHVSKEEQ
D20	RVHVSKEEQYYDYE
D21	KEEQYYDYEDATFE
D22	YDYEDATFETYALT
D23	ATFETYALTGISIC
D24	YALTGISICNPGSS
E 1	LTGISICNPGSSLC
E 2	
E 3	MLINNKKLLHHILP
E 4	KKLLHHILPILVLA

E 5	HILPILVLALLGMR
E 6	LVLALLGMRTAQAV
E 7	LGMRTAQAVAPGIV
E 8	AQAVAPGIVIPPKA
E 9	PGIVIPPKALFTQQ
E10	PPKALFTQQGGAYG
E11	FTQQGGAYGRCPNG
E12	GAYGRCPNGTRALT
E13	CPNGTRALTVAELR
E14	RALTVAELRGNAEL
E15	AELRGNAELQTYLR
E16	NAELQTYLRQITPG
E17	TYLRQITPGWSIYG
E18	ITPGWSIYGLYDGT
E19	SIYGLYDGTYLGQA
E20	YDGTYLGQAYGGII
E21	LGQAYGGIIKDAPP
E22	GGIIKDAPPGAGFI
E23	DAPPGAGFIYRETF
E24	AGFIYRETFCITTI
F 1	RETFCITTIYKTGQ
F 2	ITTIYKTGQPAADH
F 3	KTGQPAADHYYSKV
F 4	AADHYYSKVTATRL
F 5	YSKVTATRLLASTN
F 6	ATRLLASTNSRLCA
F 7	ASTNSRLCAVFVRD
F 8	RLCAVFVRDGQSVI
F 9	FVRDGQSVIGACAS
F10	QSVIGACASPYEGR
F11	ACASPYEGRYRDMY
F12	YEGRYRDMYDALRR
F13	RDMYDALRRLLYMI
F14	ALRRLLYMIYMSGL
F15	LYMIYMSGLAVRVH
F16	MSGLAVRVHVSKEE
F17	VRVHVSKEEQYYDY
F18	SKEEQYYDYEDATF
F19	YYDYEDATFQTYAL
F20	DATFQTYALTGISL
F21	TYALTGISLCNPAA
F22	LTGISLCNPAASIC
F23	
F24	MLRRFPTRTTAPGQ
G 1	PTRTTAPGQGGARR
G 2	APGQGGARRSRVRA
G 3	GARRSRVRALAWLL
G 4	RVRALAWLLASGAM
G 5	AWLLASGAMTHLSP
G 6	SGAMTHLSPALADV

G 7	HLSPALADVPYVLV
G 8	LADVPYVLVKTNMV
G 9	YVLVKTNMVVTSVA
G10	TNMVVTSVAMKPYE
G11	TSVAMKPYEVTPTR
G12	KPYEVTPTRMLVCG
G13	TPTRMLVCGIAAKL
G14	LVCGIAAKLGAAAS
G15	AAKLGAAASSPDAH
G16	AAASSPDAHVPFCF
G17	PDAHVPFCFGKDLK
G18	PFCFGKDLKRPGSS
G19	KDLKRPGSSPMEVM
G20	PGSSPMEVMLRAVF
G21	MEVMLRAVFMQQRP
G22	RAVFMQQRPLRMFL
G23	QQRPLRMFLGPKQL
G24	RMFLGPKQLTFEGK
H 1	PKQLTFEGKPALEL
H 2	FEGKPALELIRMVE
H 3	ALELIRMVECSGKQ
H 4	LIRMVECSGKQDCP
H 5	
H 6	MQRQAGLPLKANPM
H 7	GLLKANPMHTIAS
H 8	ANPMHTIASILLSV
H 9	TIASILLSVLGIYS
H10	LLSVLGIYSPADVA
H11	GIYSPADVAGLPTH
H12	ADVAGLPTHLYKNF
H13	LPTHLYKNFTVQEL
H14	YKNFTVQELALKLK
H15	VQELALKLKGKNQ-E
H16	LKLKGKNQEFCLTA
H17	KNQEFCLTAFMSGR
H18	CLTAFMSGRSLVRA
H19	MSGRSLVRACLSDA
H20	LVRACLSDAGHEHD
H21	LSDAGHEHDTWFDT
H22	HEHDTWFDTMLGFA
H23	WFDTMLGFAISAYA
H24	LGFAISAYALKSRI
I 1	SAYALKSRIALTVE
I 2	KSRIALTVEDSPYP
I 3	LTVEDSPYPGTPGD
I 4	SPYPGTPGDLLELQ
I 5	TPGDLLELQICPLN
I 6	LLELQICPLNGYCE
I 7	QEVRKYFCV
I 8	GYPKDGNAFNNLD

Figure S1. List of overlapping 15-mer peptides derived from all subunits of *Bordetella pertussis* toxin (PTx) and analyzed by SPOT-synthesis. Peptides were designed to comprehensively cover the full-length sequences of each toxin subunit, with sequential overlap to ensure continuous epitope mapping. Each peptide corresponds to a defined position within its respective subunit and was synthesized on cellulose support for antibody-binding analysis. This approach enabled the systematic identification of linear B-cell epitopes across the entire pertussis toxin complex.

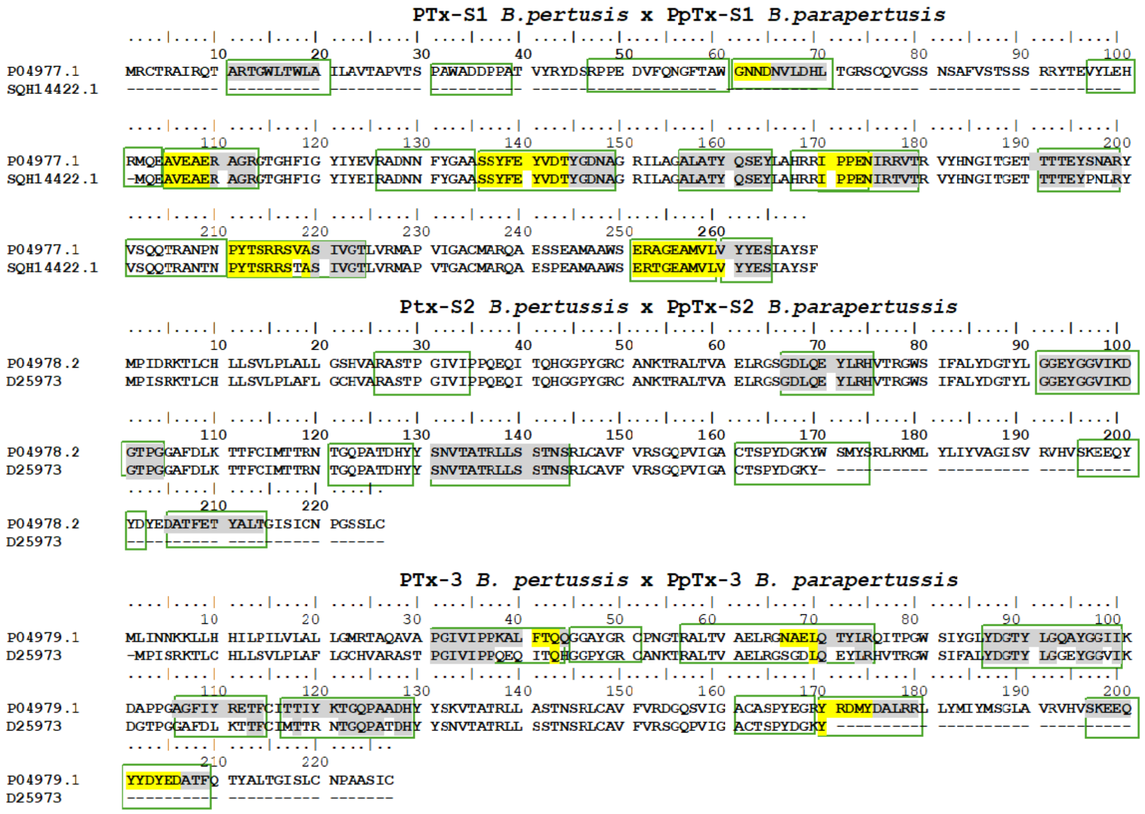

Figure S2. Homology analysis of pertussis and parapertussis toxins, demonstrating the similarity of the epitopes detected in the Ptx(s) subunits. For toxins subunit 4 and 5, *B. pertussis* lacks a toxin homologous to that of *B. parapertussis*, and no other human pathogen harbors a toxin with substantial similarity

Table S1. UniProt-reviewed bacterial toxins and adhesins were analyzed for potential epitope cross-reactivity. The dataset comprises the following UniProt entries: P01555, P01556, P43530, P32890, P08191, P13430, P43261, Q5HD51, Q53653, and Q53654. The IEDB epitope similarity analysis shows the percentage of protein sequence matches to known epitopes at an identity ≤50%.

Epitope name	Epitope Sequence	Epitope length	Percent of protein sequence matches at identity <= 100%	Minimum identity	Maximum identity	Protein ID
Ep1	ARTGWLTWLA	10	0.00% (0/10)	30.00%	40.00%	
Ep2	PAWADDPPA	9	10.00% (1/10)	22.22%	55.56%	Q53653
Ep3	SRPPEDVFQNGF TAWGNNDNVL DHL	25	0.00% (0/10)	12.00%	28.00%	
Ep4	VYLEHRMQEAV EAERAGRGTGH	22	0.00% (0/10)	13.64%	36.36%	
Ep5	RADNNFYGAAS SYFEYVDTYGDN A	24	0.00% (0/10)	16.67%	29.17%	

Ep6	ALATYQSEY	9	0.00% (0/10)	33.33%	44.44%	
Ep7	AHRRIPPENIRRVT	14	0.00% (0/10)	21.43%	42.86%	
Ep8	TTTEYSNAR	9	0.00% (0/10)	33.33%	44.44%	
Ep9	VSQQTRANPNPYTSRRSVASIVGT	24	0.00% (0/10)	16.67%	29.17%	
Ep10	ERAGEAMVLYYES	13	0.00% (0/10)	23.08%	38.46%	
Ep11	RASTPGIVI	9	20.00% (2/10)	22.22%	55.56%	P43261/Q5HD51
Ep12	GDLQEYLRH	9	0.00% (0/10)	22.22%	44.44%	
Ep13	GGEYGGVIKDGTPG	14	0.00% (0/10)	21.43%	35.71%	
Ep14	TGQPATDHY	9	0.00% (0/10)	33.33%	44.44%	
Ep15	SNVTATRLLSSTNS	14	0.00% (0/10)	21.43%	42.86%	
Ep16	CTSPYDGKYWSMYS	14	0.00% (0/10)	21.43%	35.71%	
Ep17	SKEEQYYD	8	20.00% (2/10)	25.00%	50.00%	P43261/Q53654
Ep18	DATFETYALT	10	10.00% (1/10)	30.00%	50.00%	Q53653
Ep19	LGMRTAQAVAPGIVIPPKAL	20	0.00% (0/10)	20.00%	30.00%	
Ep20	PGIVIPPKALFTQQGGAYGRC	21	0.00% (0/10)	19.05%	28.57%	
Ep21	RALTVAELRGNAELQTYLR	19	0.00% (0/10)	21.05%	31.58%	
Ep22	YDGTYLGQAYGGII	14	0.00% (0/10)	28.57%	42.86%	
Ep23	AGFIYRETF	9	0.00% (0/10)	33.33%	44.44%	
Ep24	ITTIYKTGQPAADH	14	0.00% (0/10)	21.43%	42.86%	
Ep25	ACASPYEGRYRDMYDALRR	19	0.00% (0/10)	21.05%	31.58%	
Ep26	SKEEQYYDYED	11	0.00% (0/10)	27.27%	36.36%	
Ep27	PTRTTAPGQ	9	0.00% (0/10)	22.22%	44.44%	
Ep28	TSVAMKPYEVTPTR	14	0.00% (0/10)	21.43%	42.86%	
Ep29	GPKQLTFEGK	10	0.00% (0/10)	20.00%	40.00%	
Ep30	ALELIRMV	8	0.00% (0/10)	25.00%	37.50%	
Ep31	LSDAGHEHDTWFDTMLGFA	19	0.00% (0/10)	21.05%	36.84%	
Ep32	LTVEDSPYPGTPGDLLEL	18	0.00% (0/10)	22.22%	44.44%	
